# RF-GANs: A Method to Synthesize Retinal Fundus Images Based on Generative Adversarial Network

**DOI:** 10.1155/2021/3812865

**Published:** 2021-11-10

**Authors:** Yu Chen, Jun Long, Jifeng Guo

**Affiliations:** Information and Computer Engineering College, Northeast Forestry University, Harbin, China

## Abstract

Diabetic retinopathy (DR) is a diabetic complication affecting the eyes, which is the main cause of blindness in young and middle-aged people. In order to speed up the diagnosis of DR, a mass of deep learning methods have been used for the detection of this disease, but they failed to attain excellent results due to unbalanced training data, i.e., the lack of DR fundus images. To address the problem of data imbalance, this paper proposes a method dubbed retinal fundus images generative adversarial networks (RF-GANs), which is based on generative adversarial network, to synthesize retinal fundus images. RF-GANs is composed of two generation models, RF-GAN1 and RF-GAN2. Firstly, RF-GAN1 is employed to translate retinal fundus images from source domain (the domain of semantic segmentation datasets) to target domain (the domain of EyePACS dataset connected to Kaggle (EyePACS)). Then, we train the semantic segmentation models with the translated images, and employ the trained models to extract the structural and lesion masks (hereafter, we refer to it as Masks) of EyePACS. Finally, we employ RF-GAN2 to synthesize retinal fundus images using the Masks and DR grading labels. This paper verifies the effectiveness of the method: RF-GAN1 can narrow down the domain gap between different datasets to improve the performance of the segmentation models. RF-GAN2 can synthesize realistic retinal fundus images. Adopting the synthesized images for data augmentation, the accuracy and quadratic weighted kappa of the state-of-the-art DR grading model on the testing set of EyePACS increase by 1.53% and 1.70%, respectively.

## 1. Introduction 

DR is a common complication of diabetes. The disease is caused by the blocking of the blood capillaries that nourish the retina when there is so much sugar in the blood, and then cutting off the blood supply to the retina. DR can be graded into 5 levels of severity (normal, mild, moderate, severe nonproliferative diabetic retinopathy (NPDR), and proliferative diabetic retinopathy (PDR)) according to the international protocol [1]. Human ophthalmologists usually identify and grade DR severity based on the number and size of different DR-related lesions. In brief, microaneurysms are the earliest clinically visible evidence of DR, which is the main lesion of mild NPDR. Moderate NPDR contains not only microaneurysms but also hard exudates and hemorrhages, but almost no soft exudates. Severe NPDR is characterized by the presence of intra-retinal microvascular abnormalities and soft exudate, and the absence of symptoms of PDR. Due to the ischemia of the retina, neovascularization is a significant factor of PDR. As DR worsens, it can cause serious effects on a person's vision or even blindness. Early detection of DR is therefore important for the protection of eyesight. But due to the limited medical resources and time consumption of DR detection, methods based on deep learning have prevailed in DR detection recently.

References [2–8] adopted convolutional neural network for DR grading and achieved certain results. However, due to the unbalanced distribution of EyePACS [9] (normal accounts for 73.48%, mild NPDR accounts for 6.96%, moderate NPDR accounts for 15.07%, severe NPDR accounts for 2.48%, and PDR accounts for 2.01%), the grading performance was not ideal. Although data augmentation (traditional data augmentation and advanced data augmentation [10,11]), oversampling (random oversampling [12], SMOTE [13], Borderline-SMOTE [14]), undersampling (Easy Ensemble [15], Balance Cascade [15]), and other traditional methods can mitigate the problem to some extent, the poor diversity of the images still limits model performance.

Generative adversarial network (GAN) [16] has taken off and become mainstream in the field of image generation since it was proposed by Goodfellow. GAN is optimized by generator *G* and discriminator *D* in mutual games to yield ideal outputs. Specifically, the generator *G* aims to generate real images to deceive the discriminator *D*, while *D* tries to distinguish between real images and generated images. DCGAN [17] extends GAN by employing transposed convolutional operations for upsampling; CGAN [18] adds labels to both the generator and discriminator to control the category of the synthesized images; WGAN [19] utilizes gradient penalty during training and improves the loss function, reducing the training difficulty of the model, and mitigating the problem of mode collapse; BigGAN [20] combines various novel techniques, and multiplies the number of parameters and batch size, which greatly boosts the performance of the model. Based on the favorable performance of GAN in image generation, we utilize GAN to synthesize retinal fundus images.

Specifically, the proposed RF-GANs is composed of two generation models, RF-GAN1 and RF-GAN2. RF-GAN1 translates images from source domain to target domain, then RF-GAN2 synthesizes retinal fundus images with Masks (The segmentation features include optic disk, vessel, microaneurysm, hemorrhages, hard exudates, and soft exudates) extracted by semantic segmentation models and DR grading labels. We adopt a two-stage generation of retinal fundus images for three reasons. Firstly, the amount of currently known DR-classification dataset is small. If we solely apply RF-GAN1 to translate other DR-classification dataset, we cannot get enough synthesized retinal fundus images for data augmentation. Secondly, there are very few retinal fundus images and Masks in the semantic segmentation datasets that RF-GAN2 cannot get fully trained with the Masks and images in the segmentation datasets. So, we must extract enough Masks with semantic segmentation models. Thirdly, if we solely train segmentation model with original segmentation datasets, the segmentation performance on EyePACS is not ideal (Especially some small lesions and vessels) because of domain differences, which in turn affects the quality of the retinal fundus images generated by RF-GAN2. So we apply RF-GAN1 for domain adaption to help segmentation model extract more accurate Masks. Training RF-GAN2 with more accurate Masks enhances the sensitivity of RF-GAN2 to lesion information and DR grading labels, thus allowing RF-GAN2 to precisely control the type and number of lesions according to the given DR grading labels when synthesizing retinal fundus images. So we use the combination of RF-GAN1 and segmentation models to extract enough Masks from EyePACS; then we use the extracted Masks, the corresponding retinal fundus images, and DR grading labels to train RF-GAN2.

The main contributions of this paper can be summarized into threefold: (1) RF-GAN1 is proposed to narrow down the domain gap between semantic segmentation datasets and EyePACS. RF-GAN1 extends CycleGAN [21] by integrating with Siamese Network (SiaNet) and adopting extra identity loss [22] to optimize. The number of the same class of SiaNet is set to 1. To the best of our knowledge, we are the first to perform domain adaption to reduce the domain differences between the semantic segmentation datasets and EyePACS to train segmentation models, providing large amount of more accurate Masks to train retinal fundus image generation model. (2) We propose the retinal fundus images generation model RF-GAN2 that precisely controls the DR severity level. RF-GAN2 employs multi-scale discriminator and two-stage generator to synthesize more realistic local details. Moreover, the model is optimized by adversarial loss, feature matching loss, perceptual loss, and classification loss simultaneously. (3) We conduct both qualitative and quantitative experiments to evaluate our method. Both the ophthalmologists' judgment and the quantitative assessment demonstrate that this method can synthesize realistic retinal fundus images. Moreover, utilizing the synthesized retinal fundus images for data augmentation, a significant improvement in DR grading model performance is observed.

## 2. Related Work

### 2.1. GAN in Medical Image Synthesis

Applying GAN to synthesize images to address the shortage of large and diverse datasets has been widely used in medical image processing. Kuang et al. [23] employed an encoder to map the latent space of benign lung nodules and malignant lung nodules to guide generator to synthesize corresponding lung CT images. Yang et al. [24] proposed an extra structure-consistency loss based on the modality of independent neighborhood descriptor to improve CycleGAN for unsupervised MR-to-CT synthesis. Zunair and Hamza [25] adopted adversarial training and transfer learning to convert normal and pneumonia chest X-ray to COVID-19 chest X-ray. Jiang et al. [26] extended CGAN by employing dual generators and dual discriminators that introduced a dynamic communication mechanism to improve CGAN to synthesize lung computed tomography (CT) images, then combined the generated lung CT images with nonpulmonary CT to get COVID-19 chest X-ray. Jin et al. [27] employed a new, richer convolutional feature enhanced dilated-gated generator (RicherDG) to synthesize 3D tumor lesions in CT images.

### 2.2. GAN in Retinal Fundus Image Synthesis

In recent years, there has been an interest in utilizing GAN to synthesize retinal fundus images. Costa et al. [28] implemented an adversarial auto encoder for the task of retinal vessel trees synthesis and then they adopted the generated vessel trees as an intermediate stage for the generation of retinal fundus images, which was accomplished with GAN. The use of adversarial auto encoder can synthesize more morphological vessel trees, increasing the diversity of generated retinal fundus images. But, they did not take the lesions into consideration when synthesizing retinal fundus images, so the lesion information is not obvious in the generated retinal fundus images. Zhao et al. [29] proposed to extend style transfer to the generator to increase the diversity of the synthesized retinal fundus images. This method was capable of synthesizing multiple images based on a single vessel tree. However, the DR severity level of the synthesized image was set the same as the input image, which would introduce noise when the synthesized images were applied to DR grading task. Diaz-Pinto et al. [30] proposed a new retinal fundus image synthesizer and a semi-supervised learning method for glaucoma assessment based on DCGAN. However, the generated images contained only peripheral part of the optic disc, and had a limited application. Niu et al. [31] utilized pathological descriptors extracted from the reference images as guidance to generate retinal fundus images. This method allowed for the manipulation of the location and number of lesions but did not allow for precise control of DR severity level. Burlina et al. [32] used two separate progressively grown generative adversarial networks (ProGANs) to synthesize referable and nonreferable age-related macular degeneration (AMD) images, respectively. This separate framework facilitated the generator to better learn the potential space of referable AMD and nonreferable AMD, improving the quality of the synthesized retinal fundus images. But, this study only generated two types of AMD retinal fundus images (referable AMD and nonreferable AMD). Yoo et al. [33] used CycleGAN to reduce artifacts in retinal fundus images to improve the image quality. But in several cases, as the artifact became more obscure, the checkerboard-like artifact became more prominent in generated images. Tavakkoli et al. [34] used a two-stage generator based on CGAN and used retinal fundus images as inputs for fluorescein angiography (FA) generation. The method could produce high-quality FA images even when the quality of their counterparts was relatively low. But, they did not perform longitudinal studies of benefits of the proposed method in evaluating disease progression. Lim et al. [35] adopted StyleGAN [36] as generator and ResNet-50 as discriminator to synthesize retinal fundus images with latent variable. They first trained the GAN model using all DR-grading-level retinal fundus images before fine-tuning with the desired high DR-grading-level retinal fundus images, and finally selecting probable synthetic images with an existing discriminator. This approach enhanced the sensitivity of the model to retinal fundus images of high DR grading level but had a reduced ability to generate normal retinal fundus images. Wang et al. [37] proposed a multichannel-based GAN (MGAN), which could generate a series of subfundus images, including effective local features, and all the subfundus images were combined to obtain the most representative feature of the entire fundus images. In this way, their method could deal with the challenge that effective DR features are diffuse in high-resolution fundus images. But, they mainly aimed to obtain a good discriminator for semi-supervised DR grading, and the generated images were not uniform with the real images sometimes. Yang et al. [38] added short connection structure in the generator and replaced convolutional layer with dense connection blocks in the discriminator for retinal vessel segmentation. The addition of short connection structure solved the problem of network degradation and gradient disappearance, which made the training of generator more stable, and the addition of dense connection blocks strengthened the transfer of features. But, the model was trained on small datasets, and the generalizability of the model was poor. Previous methods of generating retinal fundus images have relied excessively on manually annotated vessel trees (They cannot generate accurate vessel trees automatically), but the existing datasets of vessel trees are small. Besides, they cannot control the DR severity level of the synthesized images. In order to compensate for the above limitations, we employ RF-GAN1 to translate images from source domain to target domain to enable semantic segmentation models extract more accurate Masks to train RF-GAN2. Then, we utilize trained RF-GAN2 to synthesize high-fidelity retinal fundus images using the Masks and DR grading labels.

## 3. Proposed Method

RF-GANs proposed in this paper consist of two generation models, RF-GAN1 and RF-GAN2. This section first introduces the overall framework of RF-GANs, and then introduces RF-GAN1 and RF-GAN2, respectively.

### 3.1. Overall Framework

**Figure 1:**
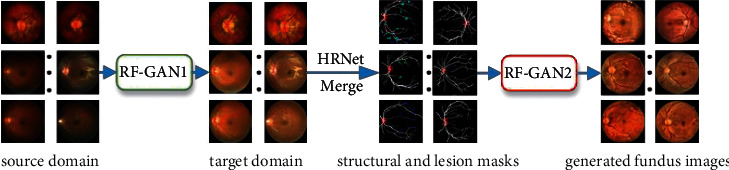
The pipeline of synthesizing retinal fundus images.

RF-GANs consists of two generation models: RF-GAN1, which aims to bridge the domain gap between the semantic segmentation datasets and EyePACS, and RF-GAN2, which is used to synthesize retinal fundus images with Masks extracted by semantic segmentation models and DR grading labels. The overall framework is shown in Figure 1.

The pipeline includes two steps; in the first step, we employ RF-GAN1 to translate retinal fundus images from source domain to target domain. Then, we adopt HR-Net [39] (HR-Net maintains a high-resolution representation by concatenating high-resolution to low-resolution convolutions in parallel, and enhances the high-resolution representation by performing multi-scale fusion repeatedly across parallel convolutions. HR-Net has achieved excellent results in human pose estimation, and we find in our experiments that HR-Net also has good performance for the segmentation of structural and lesion information in retinal fundus images) to extract the Masks of images in EyePACS. In the second step, we apply RF-GAN2 to synthesize retinal fundus images with Masks and DR grading labels.  Algorithm 1 gives the details of our proposed methods.

### 3.2. RF-GAN1

Two tailored requirements for RF-GAN1 need to be met during translating: (1) The translated datasets have a similar style to EyePACS. (2) The structures (optic disc, fundus blood vessels, etc.) and lesions (microaneurysms, hard exudates, soft exudates, hemorrhages, etc.) of the retinal fundus images remain unchanged after translating. Based on the above discussions, RF-GAN1 needs to meet the two constraints of style transfer and retention of detailed information, so the loss function of RF-GAN1 is

(1) $$\begin{array}{c} {ℒ}_{\text{RF}-\text{GAN}1}={ℒ}_{\text{Style}}+\lambda {ℒ}_{\text{Info}}, \end{array}$$

where ℒ_Style_ denotes style transfer loss, ℒ_Info_ denotes information retention loss, and *λ* is the balance parameter between the two losses.

Since the images from semantic segmentation datasets and the images from EyePACS are not paired, the style transfer can be regarded as an unpaired image-to-image transfer task. Due to the excellent performance of CycleGAN in unpaired image-to-image transfer task, RF-GAN1 is extended on the basis of CycleGAN. Suppose *G* denotes the generator from the source domain *S* to the target domain *T*, *F* denotes the generator from the target domain *T* to the source domain *S*, *D*_*S*_ denotes the discriminator of the source domain *S*, and *D*_*T*_ denotes the discriminator of the target domain *T*. The loss function of style transfer is

(2) $$\begin{array}{c} {ℒ}_{\text{Style}}={ℒ}_{\text{Adv}}\left(F,D_{S},T,S\right)+{ℒ}_{\text{Adv}}\left(G,D_{T},S,T\right)+\lambda_{1}{ℒ}_{\text{cyc}}\left(G,F\right)+\lambda_{2}{ℒ}_{\text{ide}}\left(G,F,S,T\right). \end{array}$$

In equation (2), ℒ_Adv_ denotes the adversarial loss, ℒ_cyc_ denotes the cycle-consistency loss, and ℒ_ide_ denotes the target domain identity constraint. ℒ_Adv_ makes the distribution of retinal fundus images generated by the generator as close to the distribution of real retinal fundus images as possible, ℒ_cyc_ indicates the direction of the style transfer of the retinal fundus images and ℒ_ide_ helps preserve the color of the translated retinal fundus images. The equations of ℒ_Adv_(*F*, *D*_*S*_, *T*, *S*), ℒ_Adv_(*G*, *D*_*T*_, *S*, *T*), ℒ_cyc_(*G*, *F*), ℒ_ide_(*G*, *F*, *S*, *T*) are

(3) $$\begin{array}{c} {ℒ}_{\text{Adv}}\left(F,D_{S},T,S\right)=E_{x_{S}∼S}\left[{\left(D_{S}\left(x_{S}\right)-1\right)}^{2}\right]+E_{x_{T}∼T}\left[D_{S}{\left(F\left(x_{T}\right)\right)}^{2}\right], \end{array}$$

(4) $$\begin{array}{c} {ℒ}_{\text{Adv}}\left(G,D_{T},S,T\right)=E_{x_{T}∼T}\left[{\left(D_{T}\left(x_{T}\right)-1\right)}^{2}\right]+E_{x_{S}∼S}\left[{\left(D_{T}\left(G\left(x_{S}\right)\right)\right)}^{2}\right], \end{array}$$

(5) $$\begin{array}{c} {ℒ}_{\text{cyc}}\left(G,F\right)=E_{x_{S}∼S}\left[{\left\|F\left(G\left(x_{S}\right)\right)-x_{S}\right\|}_{1}\right]+E_{x_{T}∼T}\left[{\left\|G\left(F\left(x_{T}\right)\right)-x_{T}\right\|}_{1}\right], \end{array}$$

(6) $$\begin{array}{c} {ℒ}_{\text{ide}}\left(G,F,S,T\right)=E_{x_{S}∼S}\left[{\left\|F\left(x_{S}\right)-x_{S}\right\|}_{1}\right]+E_{x_{T}∼T}\left[{\left\|G\left(x_{T}\right)-x_{T}\right\|}_{1}\right]. \end{array}$$

In equations (3)–(6), denotes the image from source domain, and denotes the image from target domain.

**Figure 2:**
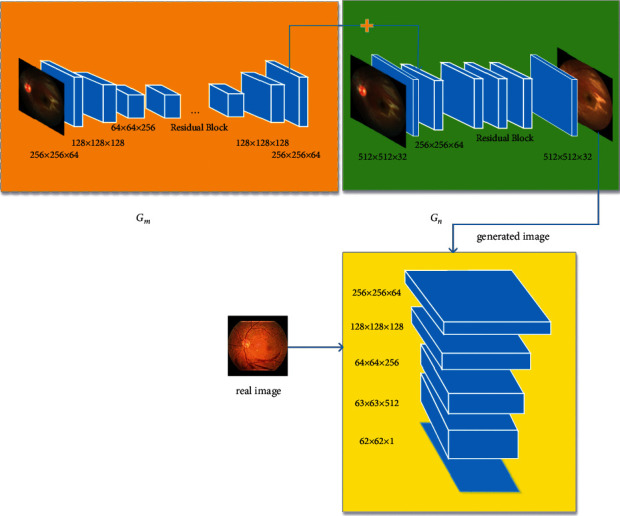
The generator and discriminator of RF-GAN1. *G*_*m*_ is the generator of the first stage, *G*_*n*_ is the generator of the second stage, and *D* is the discriminator.

Due to the high-resolution requirements of medical images, RF-GAN1 changes the generator of CycleGAN to two-stage coarse-to-fine generator [40]. The structures of generator and discriminator of RF-GAN1 are shown in Figure 2.

As illustrated in Figure 2, *G*_*m*_ is the generator of the first stage, of which inputs are images of size 256 × 256. It is composed of encoding blocks, residual blocks, and decoding blocks. The structure of *G*_*m*_ is exactly identical to that of CycleGAN except that the number of residual blocks is nine and there is no 7 × 7 convolutional operation at the last layer of the decoding block. The final outputs of *G*_*m*_ are feature maps with shape of 256 × 256 × 64. In the second stage, the inputs of *G*_*n*_ are images of size 512 × 512. *G*_*n*_ consists of encoding blocks, residual blocks, and decoding blocks. In the encoding blocks, the kernel size of the first convolutional layer is 7, and the kernel size of the second convolutional layer is 3. We configure the convolutional operations with a stride of 2 to replace pooling operations. In order to enable *G*_*n*_ to inherit the global features learned by *G*_*m*_, the inputs of the first residual block of *G*_*n*_ are the element-wise sum of the outputs of the second convolutional layer of *G*_*n*_ and the outputs of the last layer of *G*_*m*_. *G*_*n*_ employs three residual blocks. The hyper-parameter settings of the transposed convolutional operations in decoding blocks are similar to that of the convolutional operations in the encoding blocks. Finally, RF-GAN1 synthesizes transferred retinal fundus images. The synthesized images and the images of target domain are input into the discriminator *D* to distinguish between the real and the synthesized. The generator and the discriminator are optimized through the adversarial process.

**Figure 3:**
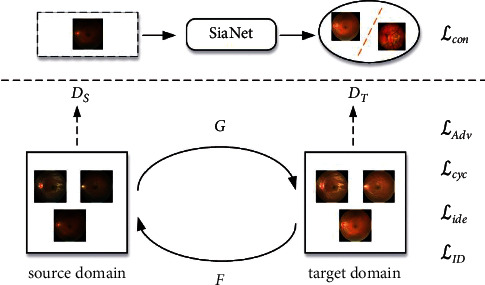
Overall framework of RF-GAN1. The upper part is SiaNet and the lower part is the generation model, which is improved on the basis of CycleGAN. The specific architecture of the generator and discriminator is shown in Figure 2.

In extensive experiments, we find that solely employing the above generator and discriminator for style transfer will lead to the loss of retinal fundus images details. To solve the issue, we integrate the generation model with SiaNet of which the number of the identical class is 1 on the one hand, and adopt identity loss to optimize the generation model on the other hand. The overall framework of RF-GAN1 is shown in Figure 3 and the upper part is SiaNet. The structure of SiaNet is shown in Table 1.

The loss function of SiaNet is Contrastive Loss [41], which helps SiaNet pull close the translated retinal fundus image and its counterpart in the source domain, and push away the translated retinal fundus image and any other retinal fundus images in the target domain. The equation of Contrastive Loss is:

(7) $$\begin{array}{c} {ℒ}_{\text{con}}\left(i,x_{1},x_{2}\right)=\left(1-i\right){\left\{\max\left(0,m-d\right)\right\}}^{2}+{\text{id}}^{2}. \end{array}$$

The equation of *d* is

(8) $$\begin{array}{c} d={\left\|\frac{x_{1}}{{\left\|x_{1}\right\|}_{2}+\varepsilon }-\frac{x_{2}}{{\left\|x_{2}\right\|}_{2}+\varepsilon }\right\|}_{2}. \end{array}$$

In equation (7), and are a pair of input vectors. *d* measures the similarity between the two input vectors *x*_1_ and *x*_2_. The calculation of *d* is illustrated in equation (8), where *ϵ* is a small nonzero constant. *i* is the binary label of the input vector pair and *x*_2_. If *x*_1_ and *x*_2_ are positive pairs, then *i* = 1. If *x*_1_ and *x*_2_ are negative pairs, then *i* = 0. We define *G*(*x*_*S*_) and *x*_*S*_, *F*(*x*_*T*_) and *x*_*T*_ as two positive vector pairs, *G*(*x*_*S*_) and *x*_*T*_, *F*(*x*_*T*_) and *x*_*S*_ as two negative vector pairs. *m* represents the classification boundary of *x*_1_ and *x*_2_. For the best performance of the model, we set *m* = 2.

In addition to the loss in equation (2), we adopt identity loss ℒ_ID_ to enhance the stability of information retention.

(9) $$\begin{array}{c} {ℒ}_{\text{ID}}=E\left[{\left\|\left(G\left(x_{S}\right)-x_{S}\right)⊙M\left(x_{S}\right)\right\|}_{2}\right], \end{array}$$

where *G* denotes the generator from source domain to target domain, *x*_*S*_ denotes the image from source domain, *M*(*x*_*S*_) denotes the Masks of *x*_*S*_, and ⊙ denotes Hadamard product. The identity loss ℒ_ID_ forces generator to preserve the local details of the retinal fundus images when performing style transfer.

Through the coordination of CycleGAN, SiaNet, and identity loss, RF-GAN1 can synthesize images which not only possess the style of target domain but also preserve local details. The loss function of RF-GAN1 is

(10) $$\begin{array}{c} {ℒ}_{\text{RF}-\text{GAN}1}={ℒ}_{\text{Adv}}\left(F,D_{S},T,S\right)+{ℒ}_{\text{Adv}}\left(G,D_{T},S,T\right)+\lambda_{1}{ℒ}_{\text{cyc}}\left(G,F\right)+\lambda_{2}{ℒ}_{\text{ide}}\left(G,F,S,T\right)+\lambda_{3}{ℒ}_{\text{con}}+\lambda_{4}{ℒ}_{\text{ID}}, \end{array}$$

where *λ*_1_, *λ*_2_, *λ*_3_, and *λ*_4_ control the weights of different losses. We set *λ*_1_=10, *λ*_2_=5, *λ*_3_=5, and *λ*_4_=2. The first three losses belong to style transfer loss, and the last two losses belong to information retention loss.

### 3.3. RF-GAN2

**Figure 4:**
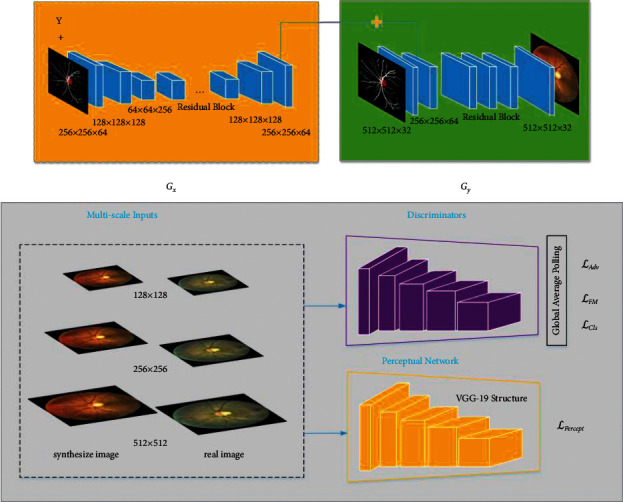
The overall architecture of RF-GAN2. The upper part is a coarse-to-fine two-stage generator, and the lower parts are multi-scale discriminator and perceptual network based on the VGG-19 backbone, respectively.

Since RF-GAN2 needs to achieve accurate control of DR severity level of synthesized images, RF-GAN2 is improved based on CGAN. The structure of RF-GAN2 is shown in Figure 4.

To synthesize high-resolution retinal fundus images, RF-GAN2 employs a two-stage coarse-to-fine generator. As shown in the upper part of Figure 4, *G*_*x*_ is the generator of the first stage, and the inputs of *G*_*x*_ are Masks of size 256 × 256 and DR grading labels *y*. *G*_*x*_ consists of encoding blocks, residual blocks, and decoding blocks. The encoding blocks employ three convolutional layers. The kernel size of the first convolutional layer is 7 and the kernel size of other two convolutional layers is 3. We configure convolutional operations with a stride of 2 to replace pooling operations for downsampling, and a ReLU and batch normalization [42] are adopted after each layer. The number of residual blocks is nine, which is used to increase the depth of the network and learn deep representations of the features of retinal fundus images. The decoding blocks consist of two transposed convolutional layers. The kernel size of the transposed convolutional layers is 3, and the hyper-parameter settings are the same as the convolutional layers in the encoding blocks. The final outputs of *G*_*x*_ are feature maps with shape of 256 × 256 × 64. *G*_*y*_ is the generator of the second stage, and the inputs of *G*_*y*_ are Masks of size 512 × 512. *G*_*y*_ is composed of encoding blocks, residual blocks, and decoding blocks. The encoding blocks employ two convolutional layers. The kernel size of the first convolutional layer is 7, and the kernel size of the second convolutional layer is 3. The remaining settings are the same as the convolutional layers in *G*_*x*_. The inputs of the first residual block of *G*_*y*_ are the element-wise sum of the outputs of the last layer of *G*_*x*_ and the outputs of the second convolutional layer of *G*_*y*_ acquires global information learned by *G*_*x*_. *G*_*y*_ employs three residual blocks. In the decoding blocks, we firstly employ a transposed convolutional layer with the kernel size of 3, and then we adopt a convolutional layer with the kernel size of 7 to synthesize retinal fundus images. Other settings are the same as the convolutional layers corresponding to the encoding blocks of *G*_*y*_.

For the purpose of differentiating between high-resolution synthesized images and real images, we employ multi-scale discriminator. Specifically, we employ three discriminators *D*_1_, *D*_2_ and *D*_3_ with the same structure, but their input image sizes are 512 × 512, 256 × 256, and 128 × 128, respectively. Although the structure of *D*_1_, *D*_2_ and *D*_3_ are identical, the coarsest scale discriminator *D*_3_ has the largest receptive field and contains more information about the global perspective of the image, which can guide the generator to synthesize holistic retinal fundus images structure and some big lesion patterns, and the finest scale discriminator *D*_1_ has the smallest receptive field which can guide the generator to synthesize more local details, particularly some small lesions.

In this paper, we devise a multi-task loss to optimize the generator and discriminator, i.e., adversarial loss, feature matching loss, perceptual loss, and classification loss. The equation of adversarial loss is

(11) $$\begin{array}{c} {ℒ}_{\text{Adv}}=\sum_{n=1,2,3}E\left[{\left(D_{n}\left(c,x\right)-1\right)}^{2}\right]+E\left[{\left(D_{n}\left(c,G\left(c,y\right)\right)\right)}^{2}\right], \end{array}$$

where *G*_*x*_ and *G*_*y*_ are collectively called *G*, *x* denotes the retinal fundus image, *n* represents images of different sizes, and *y* denotes the DR grading label of the retinal fundus image. And, *c* denotes different scale Masks input to *G*_*x*_ and *G*_*y*_, we fuse them into one conditional map denoted as *c*. In order to enhance the stability of training, we adopt feature matching loss ℒ_Feat_match_. Specifically, we extract features from the middle layers of the discriminators and make the generator match the intermediate representations between real retinal fundus images and synthesize retinal fundus images. Feature matching loss ℒ_Feat_match_ can provide more accurate guidance to the generator, enhancing the medical fidelity of the generated retinal fundus images. The equation of feature matching loss is

(12) $$\begin{array}{c} {ℒ}_{\text{Feat}\_\text{match}}=\sum_{n=1,2,3}E\left(\left[{\left\|D_{n}^{p}\left(c,x\right)-D_{n}^{p}\left(c,G\left(c,y\right)\right)\right\|}_{1}\right]\right), \end{array}$$

where *n* denotes images of different sizes, and *p* denotes the *p*^th^ intermediate layer of the discriminator, *p* ∈ [1,5]. Due to the excellent performance of perceptual loss on super resolution generation and style transfer, we employ perceptual loss ℒ_Percept_ to optimize the discriminator and generator. In such a context, using perceptual loss can enhance the local details of the synthesized retinal fundus images. The equation of perceptual loss is

(13) $$\begin{array}{c} {ℒ}_{\text{Percept}}=\sum_{n=1,2,3}E\left(\left[{\left\|F^{q}\left(x\right)|-|F^{q}|\left(G\left(c,y\right)\right)\right\|}_{1}\right]\right), \end{array}$$

where *n* denotes images of different sizes, *F* denotes perceptual network based on VGG-19 [43] backbone, *q* denotes the *q*^th^ intermediate layer of the perceptual network, *qϵ*[1,5]. We adopt classification loss to precisely control the DR severity level of the synthesized retinal fundus images, so that the generator can control the type and number of lesions according to the DR grading labels. The equation of classification loss is:

(14) $$\begin{array}{c} {ℒ}_{\text{Cls}}=\sum_{n=1,2,3}{ℒ}_{\text{CE}}\left(D_{n}\left(x\right),y\right)+{ℒ}_{\text{CE}}\left(D_{n}\left(G\left(c,y\right)\right),y\right), \end{array}$$

where ℒ_CE_ denotes cross entropy loss. Combining the above losses, the loss function of RF-GAN2 is

(15) $$\begin{array}{c} {ℒ}_{\text{RF}-\text{GAN}2}={ℒ}_{\text{Adv}}+\lambda_{1}{ℒ}_{\text{Feat}\_\text{match}}+\lambda_{2}{ℒ}_{\text{Percept}}+\lambda_{3}{ℒ}_{\text{Cls}}, \end{array}$$

where *λ*_1_, *λ*_2_, and *λ*_3_ denote the weights of different losses. We set *λ*_1_=5, *λ*_2_=5, *λ*_3_=1.

## 4. Experiments and Results

### 4.1. Datasets and Preprocessing

In this paper, we mainly exploit five datasets: EyePACS, FGADR [44], IDRiD [45], DRIVE [46], and a private DR grading dataset provided by a local hospital (We call it private dataset). EyePACS is exploited to acquire Masks so that RF-GAN2 can synthesize retinal fundus images with them and can be used as training and testing set of DR grading models. The Seg-set of FGADR is employed to train the lesion segmentation models, and the Grade-set of FGADR is employed to evaluate DR grading models. IDRiD is employed to train the optic disc segmentation model. DRIVE is adopted to train the fundus vessel segmentation model. The private dataset is adopted to evaluate the performance of the DR grading models. The detailed introduction about the five datasets is given below.

  EyePACS : The dataset is provided by EyePACS, containing 35126 training images and 53576 testing images, all of which only have DR grading labels.
  FGADR : The dataset consists of a Grade-set which contains 1000 retinal fundus images with only DR grading labels and a Seg-set, which contains 1842 retinal fundus images with pixel-level annotations of lesions—microaneurysms, hard exudates, soft exudates, hemorrhages, etc.
  IDRiD : The dataset contains a total of 81 images, each with pixel-level annotations of the optic disc and DR lesions, such as microaneurysms, hard exudates, soft exudates, hemorrhages.
  DRIVE : A total of 40 images are included in the dataset, 33 of which do not show any signs of diabetic retinopathy while 7 of which show signs of mild diabetic retinopathy. Each image has pixel-level annotations of fundus vessels.
  Private dataset : The dataset is collected from a local hospital and labeled by three ophthalmologists. The private dataset has 2758 retinal images with different criteria for DR grades 0 to 4.

For the purpose of enhancing the contrast of the retinal fundus images in EyePACS to improve the accuracy of DR grading, we conduct contrast limited adaptive histogram equalization (CLAHE) on the retinal fundus images. The number of images in FGADR, IDRiD, and DRIVE datasets is very small compared with EyePACS, which harms the performance of domain adaption. We conduct clipping, flipping, rotation on FGADR, IDRiD, and DRIVE datasets, and undersample EyePACS.

### 4.2. Experiments and Results of RF-GAN1

In this section, semantic segmentation datasets (IDRiD, DRIVE and FGADR) are adopted as source domain, and EyePACS as target domain. We employ RF-GAN1 to translate images from source domain to target domain, and utilize the translated images to train HR-Net. Then, we apply the trained HR-Net (We adopt U-Net [47], PSPNet [48], DeepLab v3 [49], and HR-Net as segmentation models, and finally we find that using HR-Net is the best for segmentation of vessels, optic discs, and various lesions) to extract Masks from EyePACS.

**Figure 5:**
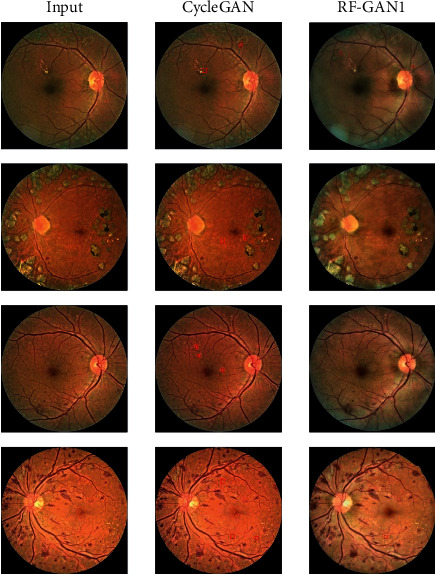
The performance demonstration of domain adaption by RF-GAN1 and CycleGAN. From left to right, they are input images from FGADR, the corresponding images translated by CycleGAN, and the corresponding images translated by RF-GAN1, respectively. Red boxes indicate local details lost after translation.

**Figure 6:**
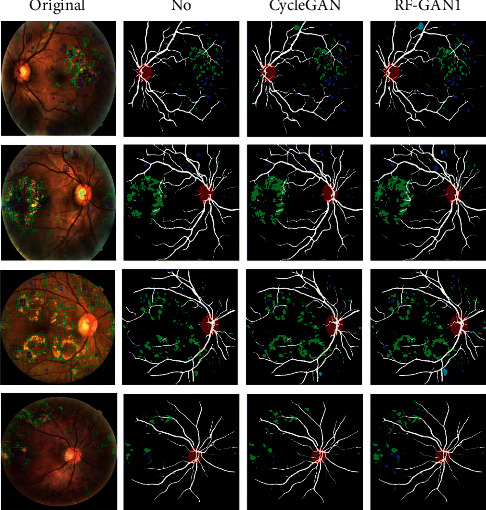
The segmentation performance of the segmentation models trained by different datasets. From left to right, they are original images from EyePACS annotated by ophthalmologists, the corresponding Masks segmented by HR-Net trained by original segmentation datasets, the corresponding Masks segmented by HR-Net trained by segmentation datasets translated by CycleGAN, and the corresponding Masks segmented by HR-Net trained by segmentation datasets translated by RF-GAN1. The white, red, green, blue, and cyan parts in the Masks indicate vessels, optic discs, hard exudates, hemorrhages, and soft exudates segmented by the segmentation models, respectively.

For visual comparisons, we employ CycleGAN to translate images from segmentation datasets to EyePACS. Then, we select 300 retinal fundus images translated by CycleGAN and RF-GAN1, respectively, and ask two professional ophthalmologists to evaluate the images. Part of the translated images is shown in Figure 5. Furthermore, we use the datasets translated by CycleGAN to train HR-Net, and apply the trained HR-Net to extract Masks. Then, we choose 300 Masks extracted by HR-Net trained by original segmentation datasets, segmentation datasets translated by CycleGAN, and segmentation datasets translated by RF-GAN1, and ask two professional ophthalmologists to evaluate the Masks, too. Part of the extracted Masks is shown in Figure 6.

We summarize the feedback of the two ophthalmologists. The first is for the evaluation of translated images. The style of images translated by CycleGAN is almost unchanged and remains similar to the style of original FGADR. Moreover, there is partial loss of fine lesion information after translation (as is illustrated in Figure 5). However, the fidelity of the retinal fundus images translated by RF-GAN1 is better. The style is similar to that of EyePACS and the local details of the images are better maintained than that of CycleGAN after translation. Although there are still errors after translation by RF-GAN1, the impact on HR-Net training is not significant.

The second is for the evaluation of Masks. HR-Net trained by the original datasets can detect relatively obvious hemorrhages and hard exudates but not soft exudates. In addition, the models are not sensitive to some small structures or lesions, such as microaneurysms, smaller areas of hard exudate, hemorrhage, and some small vessels (as shown in the second column of Figure 6, only a proportion of obvious hemorrhages and hard exudates are extracted, but most lesions, as well as fine vessels, are not extracted by HR-Net). HR-Net trained by datasets translated by CycleGAN are more sensitive to small areas of hard exudates compared to the models trained directly by original datasets, but still fails to segment soft exudates as well as microaneurysms and some fine vessels (as shown in the third column of Figure 6, we can see some fine areas of hard exudates, but can hardly notice microaneurysms, soft exudates, and some fine vessels). The segmentation performance of HR-Net trained by datasets translated by RF-GAN1 is better. The models can be successful in segmenting fine areas of hard exudates and hemorrhages, as well as soft exudates. Besides, the models are more sensitive to microaneurysms and small vessels (as shown in the fourth column of Figure 6, HR-Net is able to extract not only obvious lesions such as hard exudates and hemorrhages but also lesions that are not easy to segment such as microaneurysms, soft exudates. In addition, HR-Net performs well for the segmentation of fine vessels). Although there are still errors in the Masks, they are within tolerable limits.

From the evaluation of ophthalmologists, we can see that RF-GAN1 is better for the style transfer of retinal fundus images to improve the segmentation performance on EyePACS. And, we analyze the following reasons for this situation. Firstly, the reason behind the poor domain adaption performance of CycleGAN might be that the model falls into the local minimum. The generator can deceive the discriminator by synthesizing images that are very close to original retinal fundus images, but the discriminator does not have enough discriminant ability to distinguish them. Secondly, the excellent domain adaption performance of RF-GAN1 is due to two reasons. (1) SiaNet can pull different fundus images away to avoid the model falling into local minimum, so as to perform style transfer favorably. (2) Plugging SiaNet and identity loss into generation model increases the model's attention to local details, so that the synthesized images can better retain the information of structures and lesions.

Since EyePACS has no pixel-level annotations, it is impossible to quantitatively evaluate the segmentation performance of the models trained by the datasets translated by RF-GAN1 on EyePACS. So in the supplemental material, we use the segmentation dataset to validate the segmentation ability of combining HR-Net with RF-GAN1 for DR-related lesions and fundus vessels to show the domain adaptation ability of RF-GAN1 for retinal fundus images.

### 4.3. Experiments and Results of RF-GAN2

In this section, we train RF-GAN2 with the Masks, and the corresponding retinal fundus images and DR grading labels until they converge. Then, we utilize Masks and DR grading labels to synthesize 10000 retinal fundus images with RF-GAN2 for each DR severity level (there are some low-quality images in the generated fundus images, but we do not process them because the number is small and have little impact on DR grading model training). In order to quantitatively assess the synthesized images, we use Frechet inception distance (FID) [50] and sliced wassertein distance (SWD) to evaluate the images. In addition, the synthesized images are added into the training set of EyePACS for data augmentation, and we evaluate the performance of the DR grading models on the testing set of EyePACS, FGADR, and private dataset.

#### 4.3.1. Performance Demonstration

**Figure 7:**
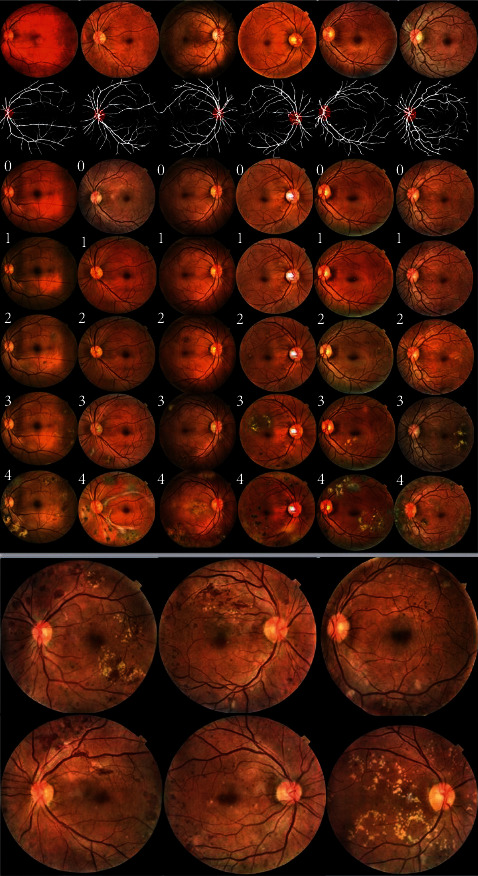
Retinal fundus images synthesized by RF-GAN2. The upper part, the first row shows the original images from EyePACS, the second row shows the Masks extracted from original images, and the subsequent rows are normal, mild, moderate, severe NPDR and PDR retinal fundus images synthesized with the Masks in the second row and DR grading labels, and the lower part is the detailed display of the synthesized images.

Part of the synthesized images is shown in Figure 7. It should be noted that the EyePACS adopted for training RF-GAN2 is processed by CLAHE, so the style of synthesized images resembles that of the EyePACS processed by CLAHE. As can be seen from the upper part of Figure 7, given Masks and DR grading labels, RF-GAN2 can synthesize high-fidelity retinal fundus images, and the appearance and number of lesions in the images can be manipulated by the DR grading labels. The lower part of Figure 7 shows the details of the synthesized images. The structural information such as optic disc, fundus vessels, macula, and the pathological information such as microaneurysms, hemorrhages, soft exudates, and hard exudates can be clearly seen.

#### 4.3.2. Comparison with Other Models

**Figure 8:**
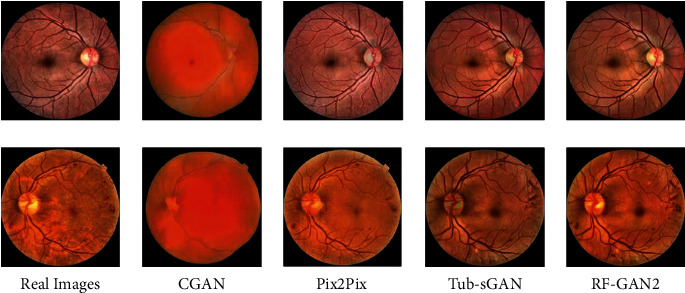
Visual comparison with other models. The first column shows original retinal fundus images from EyePACS, the second to fourth columns show retinal fundus images synthesized by CGAN, Pix2Pix, Tub-sGAN, and RF-GAN2, respectively.

FID is a commonly used image quality evaluation metric, which is a measure to calculate the distance of feature vectors between the real images and the synthesized images. FID is calculated by computing the Frechet distance of the Gaussian distribution constructed by features extracted from the real and synthesized images in Inception-v3 [51]. The lower the score, the higher the similarity between the synthesized images and the real images, and the better the image quality. SWD is also a commonly used image quality evaluation metric. SWD is usually employed to evaluate the quality of high-resolution images. It is calculated by evaluating the statistical similarity of real images and synthesized images. The lower the SWD, the higher the image quality. We quantitatively evaluate the retinal fundus images synthesized by RF-GAN2, CGAN, Pix2Pix [52], and Tub-sGAN [29] with FID and SWD. The results are shown in Table 2 and the synthesized images by different models are shown in Figure 8.

From Table 2, we can see that retinal fundus images synthesized by RF-GAN2 achieve a FID score of 7.03 and a SWD score of 11.28, which is better than Tub-sGAN and other baseline models. From the visual perspective of Figure 8, two ophthalmologists make the following comments on the synthesized retinal fundus images by different models. The retinal fundus images synthesized by CGAN can only see the fundus outline and some of the main fundus vessels, and cannot see detailed information of structures and lesions. Pix2Pix can synthesize fundus vessels and lesion information but cannot generate fine fundus vessels and clear macular. The retinal fundus images generated by Tub-sGAN are generally satisfactory, but sometimes the macular regions are less precise. RF-GAN2 is superior to the previous three methods in that it can produce retinal fundus images with clear structural information, such as fine fundus vessels and macular areas, as well as realistic lesion information, such as hemorrhages and hard exudates.

There are several reasons behind RF-GAN2's superiority to other models. Firstly, training HR-Net with the segmentation datasets translated by RF-GAN1 improves the segmentation performance of HR-Net on EyePACS. Therefore, we obtain more accurate Masks to train RF-GAN2, increasing RF-GAN2's sensitivity to lesions. Secondly, RF-GAN2 employs two-stage generator, from coarse to fine. The second-stage generator inherits the global features extracted by the first-stage generator to further synthesize local details, so that the image quality is higher. Finally, the introduction of classification loss can control the category and number of lesions according to DR grading labels, synthesizing more diverse retinal patterns.

#### 4.3.3. Data Augmentation by Synthesis for DR Grading

**Figure 9:**
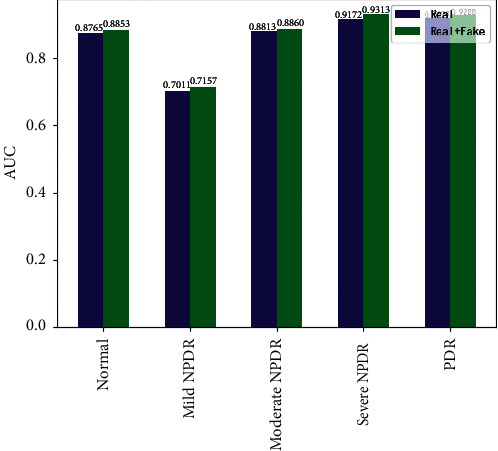
AUC of ResNet-50 for each DR severity with and without synthesis. Real denotes original retinal fundus images in the testing set of EyePACS, Fake denotes 50000 synthesized retinal fundus images.

This section verifies whether adding the synthesized images into training set for data augmentation can be beneficial for training DR grading models. We train 3 baseline models (VGG-19, ResNet-50 [53], Inception-v3), two competitive DR grading models AFN [54] and Zhou et al. (DenseNet-121) [44] with and without data augmentation by synthesized images. We only modify the output dimension of the last fully connected layer to 5 for baseline models. We employ classification accuracy and quadratic weighted kappa for evaluation. We test them on the testing set of EyePACS, and the experimental results are shown in Table 3. In order to evaluate the generalization ability of the grading models, we test the trained models on FGADR and our private dataset, and the results are shown in Table 4.  Table 5 shows the classification accuracy of different DR severity levels with ResNet-50 when testing on the testing set of EyePACS with and without data augmentation by synthesis.  Figure 9 illustrates the AUC (For each DR severity, we view the current DR severity level as positive class and the other severity levels as negative classes) of ResNet-50 for each DR severity with and without data augmentation by synthesis.

In Tables 3–5, Fake denotes 50000 synthesized images. Table 3 shows that the accuracy and quadratic weighted kappa of each DR grading model on the testing set of EyePACS increase on average by 1.63% and 1.82%, respectively. The accuracy and quadratic weighted kappa of competitive DR grading models AFN and Zhou (DenseNet-121) increase by 1.49%, 1.68% and 1.53%, 1.70%, respectively, proving the effectiveness of our method. In order to assess the generalization ability of the trained models, we test the model on the private dataset and Grade-set of FGADR (The classification models are not fine-tuned on these two datasets before). As shown in Table 4, we find that the accuracy and quadratic weighted kappa increase on average by 1.24%, 1.35% and 1.04%, 1.13% on private dataset and Grade-set of FGADR, respectively, when adding synthesized images into training set for data augmentation, which proves the generalization ability of the DR grading models trained by synthesis. As can be seen from Table 5, the classification accuracy of each DR severity level of ResNet-50 increases by 1.35%, 1.73%, 1.43%, 2.38%, and 1.78%, respectively. Similarly, in Figure 9, the AUC of each DR severity level of ResNet-50 increases by 0.0088, 0.0146, 0.0047, 0.0141, and 0.0089, respectively. The accuracy and AUC of each DR severity level increases, especially for high severity level, where there are not many images. Besides, the increase in AUC for mild NPDR is also significant, and we believe that the data augmentation by synthesis allows DR grading model to learn more about the distinguishable features between mild NPDR and normal retinal fundus images, which leads to an increase in AUC. Based on the above experiments, we can conclude that RF-GANs can synthesize realistic retinal fundus images and be beneficial for training DR grading models.

## 5. Discussion

### 5.1. Training Stability

**Figure 10:**
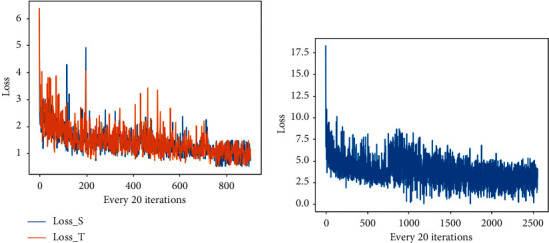
Loss curve of RF-GAN1 (a) and RF-GAN2 (b). Loss_S and Loss_T in Figure 10(a) indicate the loss curve of the generator *F* and the generator *G* in RF-GAN1, respectively.

**Figure 11:**
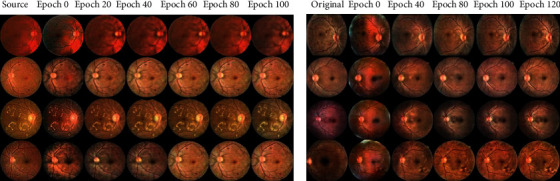
Illustrations of the synthesized images from different epochs by RF-GAN1 (a) and RF-GAN2 (b).

Since there are many loss functions applied in RF-GAN1 and RF-GAN2, we would like to plot the loss curve and show the images generated from different epochs to check the stability of the model training.  Figure 10 shows the loss curve of RF-GAN1 (Figure 10(a)) and RF-GAN2 (Figure 10(b)), and we plot points every 20 iterations.  Figure 11 illustrates the synthesized retinal fundus images from different epochs by RF-GAN1 (Figure 11(a)) and RF-GAN2 (Figure 11(b)). Figure 10(a) shows the loss curve of translating images from FGADR to EyePACS, and other two loss curves of RF-GAN1 (DRIVE to EyePACS and IDRiD to EyePACS) is similar to Figure 10(a). It can be seen from Figure 10(a) that the model fluctuates a lot at the beginning, and the model converges after about 14000 iterations, but still fluctuates a little after convergence. As can be seen from Figure 11(a), the global features, including optic disc, macula, and blood vessels in the whole fundus, are almost translated at 20th epoch (About 4000 iterations). Small lesions and structures, such as hemorrhages and fine vessels, are transferred at 60th epoch (About 12000 iterations). After 80 epochs (About 16000 iterations), the fundus images basically complete translation, at which point the model converges. From Figure 10(b), we can see that the model training is unstable, and the model converges after 40,000 iterations, but still has some fluctuations. As can be seen from Figure 11(b), the synthesized images get global styling feature and components at 40th epoch (About 16000 iterations), such as the relative position of the optic disc, vessels. At the 80th epoch (About 32000 iterations), some low-quality pictures appear, such as abnormal macular areas and loss of fine vessels, which indicate the fluctuations of RF-GAN2. From 100 to 120 epochs (About 40000 to 50000 iterations), the generated fundus images gradually stabilize and the model begins to converge. We can see that the application of a large number of loss functions is likely to cause instability during model training, which is unavoidable. However, the loss functions applied by RF-GAN1 and RF-GAN2 are beneficial to increase the accuracy and diversity of the generated retinal fundus images. So we choose to use multi-loss functions and carefully monitor the synthesized images and loss curves.

### 5.2. Diversity

**Figure 12:**
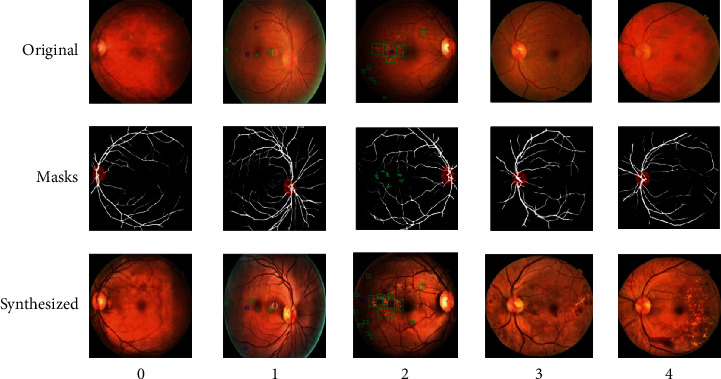
Examples of generating retinal fundus images from the original images. The blue boxes and green boxes indicate the lesions of hemorrhages and hard exudates, respectively.

We question the use of Masks and DR grading labels to generate retinal fundus images to increase the diversity of the dataset. Specifically, we want to know whether the generated retinal fundus images are identical to the original images, so we compare the synthesized images with the original images. As shown in Figure 12, we choose some examples of generating retinal fundus images from the original images to verify the diversity of the synthesized images. In Figure 12, they are original images, the corresponding Masks extracted by the trained HR-Net and the synthesized images from top to bottom. The DR severity of the synthesized images is from 0 to 4 from left to right. Both the original and generated images in the first column are normal retinal fundus images, and we observe that the synthesized images have clearer detail information than the original images, which is very helpful for DR grading. The DR severities of the original and synthesized images in the second and third columns are mild NPDR and moderate NPDR, respectively. To facilitate observation, we mark the lesions with bounding boxes in the original images and the generated images, respectively. We observe that the synthesized images have essentially the same structural information as the original images. Moreover, we observe that the categories of lesions remain essentially the same, but the number and appearances of different lesions and locations change, which increases the diversity of the dataset while ensuring that the DR severity of the synthesized images remains unchanged. The fourth and fifth columns show that we use the Masks of normal retinal fundus images to synthesize severe NPDR and PDR retinal fundus images, respectively. We can see that although neither the original retinal fundus images nor the Masks contain lesion information, we can still generate retinal fundus images of the corresponding DR severity level by specifying the DR grading labels when synthesizing them. From the above examples, we can see that our method is capable of synthesizing visually different retinal fundus images conditioned on the given DR grading labels and Masks, increasing the diversity of the dataset.

### 5.3. Limitations

**Figure 13:**
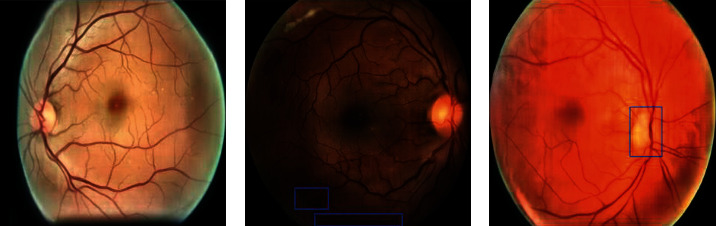
Three main failure cases generated by our method. Blue boxes indicate the failures.

Although our approach is capable of synthesizing high-quality retinal fundus images, there are still some limitations that need to be further addressed.  Figure 13 illustrates three main kinds of failure cases observed in the synthesized images.  Figure 13(a) shows that the image is not sufficiently round. Since the shape of the generated retinal fundus image is influenced by the vessel trees obtained by segmentation models, if the distribution of the vessel in the Masks is not rounded enough, then the generated retinal fundus images may also be affected.  Figure 13(b) shows the case where the structures and lesions' information of the image cannot be clearly seen due to low illumination (As shown in the bottom part of Figure 13(b)). Although we use CLAHE to enhance the contrast of fundus images in EyePACS, some low-quality images are still present. Influenced by these images, RF-GAN2 occasionally generates low-quality retinal fundus images like Figure 13(b). In Figure 13(c), the boundary of the optic disc is not clear and this is due to two reasons. On the one hand, the optic disc mask we obtain during segmentation is not precise enough and thus affects the shape of the optic disc in the generated fundus images. On the other hand, the boundary of the optic disc in the original fundus image is not clear, so the generator transforms the part which is not the optic disc into the optic disc part in the generated image. In addition to the above problems in generating images, the two-stage generation of retinal fundus images by this method is also somewhat cumbersome, but this ensures that we can generate high-quality retinal fundus images.

## 6. Conclusion

This paper proposes a retinal fundus image generation method RF-GANs based on generative adversarial network, which consists of RF-GAN1 and RF-GAN2. RF-GAN1 translates images from source domain to target domain to improve the structures and lesions' segmentation performance of HR-Net for the images from EyePACS. RF-GAN2 employs the Masks and DR grading labels to synthesize retinal fundus images. Experiments show that HR-Net trained by translated datasets get better segmentation performance for structures and lesions related to DR on EyePACS, and the retinal fundus images synthesized by RF-GAN2 are superior to the mainstream generation model and Tub-sGAN in FID and SWD. When adding the synthesized images into training set for data augmentation, the accuracy and quadratic weighted kappa of DR grading models (VGG-19, ResNet-50, Inception-v3, AFN, Zhou (DenseNet-121)) on the testing set of EyePACS increase on average by 1.63% and 1.82%, respectively. We also demonstrate the generalization ability of the models by using Grade-set of FGADR as well as the private dataset to test the trained DR grading models. Besides, the accuracy and AUC of each DR severity level on ResNet-50 all improves. The results show that RF-GANs hold promise for the future. However, we also notice that mild NPDR images are often misclassified as normal images because they are close to normal retinal fundus images, which significantly reduce the classification accuracy. In future work, we will explore how to enhance the sensitivity of DR grading models for mild NPDR retinal fundus images by GAN.

## Figures and Tables

**Algorithm 1:**
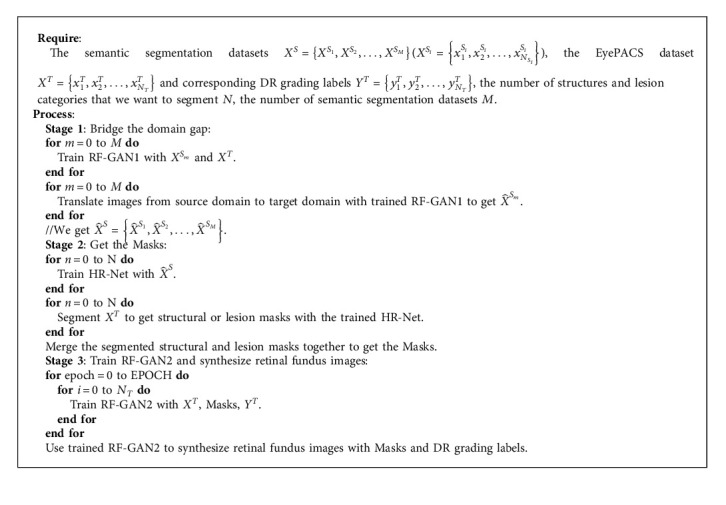
The algorithm of RF-GANs.

**Table 1:** The structure of SiaNet.

Input	Layer	Output
(512, 512, 3)	Conv (64,4 × 4), LReLU, pool	(128, 128, 64)
(128, 128, 64)	Conv (64,4 × 4), LReLU, pool	(32, 32, 128)
(32, 32, 128)	Conv (64,4 × 4), LReLU, pool	(8, 8, 256)
(8, 8, 256)	Conv (64,4 × 4), LReLU, pool	(2, 2, 512)
(1, 2048)	FC (2048 × 128), LReLU, dropout	(1, 128)
(1, 128)	FC (128 × 64), normalize	(1, 64)

**Table 2:** FID and SWD evaluation of synthesized retinal fundus images.

	AVG.FID	AVG. SWD × 10^3^
CGAN	19.45	31.94
Pix2Pix	15.24	27.92
Tub-sGAN	9.67	15.92
RF-GAN2	7.03	11.28

**Table 3:** The classification results with and without data augmentation by synthesis on the testing set of EyePACS.

Training set	EyePACS (%)		EyePACS + Fake (%)	
Metric	Acc	Kappa	Acc	Kappa
VGG-19	84.95	82.15	86.49	83.85
ResNet-50	86.27	83.86	88.07	85.91
Inception-v3	85.67	83.34	87.44	85.29
AFN	87.53	85.69	89.02	87.37
Zhou	89.46	88.48	90.99	90.18

Zhou: the method of Zhou (DenseNet-121).

**Table 4:** The classification results with and without data augmentation by synthesis on private dataset and Grade-set of FGADR.

Testing set	Private dataset (%)				FGADR grade-set (%)			
Training set	EyePACS		EyePACS + Fake		EyePACS		EyePACS + Fake	
Metrics	Acc	Kappa	Acc	Kappa	Acc	Kappa	Acc	Kappa
VGG-19	82.13	75.83	83.38	77.19	81.48	75.53	82.51	76.67
ResNet-50	83.25	76.72	84.43	78.01	82.91	76.42	83.93	77.69
Inception-v3	82.63	76.12	83.87	77.48	81.95	75.82	82.96	76.88
AFN	84.22	77.92	85.47	79.27	83.78	77.50	84.86	78.53
Zhou	86.43	82.12	87.70	83.52	85.92	84.72	87.00	85.89

Zhou: the method of Zhou (DenseNet-121).

**Table 5:** The classification accuracy (%) of ResNet-50 for each DR severity level.

	EyePACS (%)	EyePACS + Fake (%)
Grade 0	93.76	95.11
Grade 1	47.27	49.00
Grade 2	76.26	77.69
Grade 3	84.12	86.50
Grade 4	84.35	86.13

## Data Availability

The private dataset used to support the findings of this study have not been made available because of patient privacy, and other datasets are from previously reported studies and datasets, which have been cited.

## Supplementary Materials

Supplementary Materials Quantitative Evaluation of RF-GAN1.

## References

[1] (2002). *Ophthalmoscopy, Dilated and E. T. D. R. S. Levels. International Clinical Diabetic Retinopathy Disease Severity Scale Detailed Table*.

[2] Wang Z., Yin Y., Shi J., Fang W., Li H., Wang X. Zoom-in-net: deep mining lesions for diabetic retinopathy detection.

[3] Quellec G., Charrière K., Boudi Y., Cochener B., Lamard M. (2017). Deep image mining for diabetic retinopathy screening. *Medical Image Analysis*.

[4] Wan S., Liang Y., Zhang Y. (2018). Deep convolutional neural networks for diabetic retinopathy detection by image classification. *Computers & Electrical Engineering*.

[5] Esfahani M. T., Ghaderi M., Kafiyeh R. (2018). Classification of diabetic and normal fundus images using new deep learning method. *Leonardo Electronic Journal of Practices and Technologies*.

[6] Qummar S., Khan F. G., Shah S. (2019). A deep learning ensemble approach for diabetic retinopathy detection. *IEEE Access*.

[7] Jiang H., Yang K., Gao M., Zhang D., Ma H., Qian W. An interpretable ensemble deep learning model for diabetic retinopathy disease classification.

[8] Liu Y.-P., Li Z., Xu C., Li J., Liang R. (2019). Referable diabetic retinopathy identification from eye fundus images with weighted path for convolutional neural network. *Artificial Intelligence in Medicine*.

[9] (2015). Kaggle diabetic retinopathy detection competition. https://www.kaggle.com/c/diabetic-retinopathy-detection.

[10] Inoue H. (2018). Data augmentation by pairing samples for images classification. https://arxiv.org/abs/1801.02929.

[11] Yun S., Han D., Chun S., Oh S. J., Yoo Y., Choe J. CutMix: regularization strategy to train strong classifiers with localizable features.

[12] Estabrooks A., Jo T., Japkowicz N. (2004). A multiple resampling method for learning from imbalanced data sets. *Computational Intelligence*.

[13] Chawla N. V., Bowyer K. W., Hall L. O., Kegelmeyer W. P. (2002). SMOTE: synthetic minority over-sampling technique. *Journal of Artificial Intelligence Research*.

[14] Han H., Wang W.-Y., Mao B.-H. Borderline-SMOTE: a new over-sampling method in imbalanced data sets learning.

[15] Xu-Ying Liu X. Y., Jianxin Wu J., Zhou Z. H. (2009). Exploratory undersampling for class-imbalance learning. *IEEE Transactions on Systems, Man, and Cybernetics, Part B (Cybernetics)*.

[16] Goodfellow I. J., Pouget-Abadie J., Mirza M. (2014). Generative adversarial networks. https://arxiv.org/abs/1406.2661.

[17] Radford A., Metz L., Chintala S. (2015). Unsupervised representation learning with deep convolutional generative adversarial networks. https://arxiv.org/abs/1511.06434.

[18] Mirza M., Osindero S. (2014). Conditional generative adversarial nets. https://arxiv.org/abs/1411.1784.

[19] Arjovsky M., Chintala S., Bottou L. (2017). Wasserstein GAN. https://arxiv.org/abs/1701.07875.

[20] Brock A., Donahue J., Simonyan K. (2018). Large scale GAN training for high fidelity natural image synthesis. https://arxiv.org/abs/1809.11096.

[21] Zhu J. Y., Park T., Isola P., Efros A. A. Unpaired image-to-image translation using cycle-consistent adversarial networks.

[22] Wei L., Zhang S., Gao W., Tian Q. Person transfer GAN to bridge domain gap for person Re-identification.

[23] Kuang Y., Lan T., Peng X., Selasi G. E., Liu Q., Zhang J. (2020). Unsupervised multi-discriminator generative adversarial network for lung nodule malignancy classification. *IEEE Access*.

[24] Yang H., Sun J., Carass A. (2020). Unsupervised MR-to-CT synthesis using structure-constrained CycleGAN. *IEEE Transactions on Medical Imaging*.

[25] Zunair H., Hamza A. B. (2021). Synthesis of COVID-19 chest X-rays using unpaired image-to-image translation. *Social Network Analysis and Mining*.

[26] Jiang Y., Chen H., Loew M., Ko H. (2020). COVID-19 CT image synthesis with a conditional generative adversarial network. *IEEE Journal of Biomedical and Health Informatics*.

[27] Jin Q., Cui H., Sun C., Meng Z., Su R. (2021). Free-form tumor synthesis in computed tomography images via richer generative adversarial network. *Knowledge-Based Systems*.

[28] Costa P., Galdran A., Meyer M. I. (2017). End-to-End adversarial retinal image synthesis. *IEEE Transactions on Medical Imaging*.

[29] Zhao H., Li H., Maurer-Stroh S., Cheng L. (2018). Synthesizing retinal and neuronal images with generative adversarial nets. *Medical Image Analysis*.

[30] Diaz-Pinto A., Colomer A., Naranjo V., Morales S., Xu Y., Frangi A. F. (2019). Retinal image synthesis and semi-supervised learning for glaucoma assessment. *IEEE Transactions on Medical Imaging*.

[31] Niu Y., Gu L., Lu F. Pathological evidence exploration in deep retinal image diagnosis.

[32] Burlina P. M., Joshi N., Pacheco K. D., Liu T. Y. A., Bressler N. M. (2019). Assessment of deep generative models for high-resolution synthetic retinal image generation of age-related macular degeneration. *JAMA ophthalmology*.

[33] Yoo T. K., Choi J. Y., Kim H. K. (2020). CycleGAN-based deep learning technique for artifact reduction in fundus photography. *Graefes Archive for Clinical and Experimental Ophthalmology*.

[34] Tavakkoli A., Kamran S. A, Hossain K. F, Zuckerbrod S. L (2020). A novel deep learning conditional generative adversarial network for producing angiography images from retinal fundus photographs. *Scientific Reports*.

[35] Lim G., Thombre P., Lee M. L., Hsu W. Generative data augmentation for diabetic retinopathy classification.

[36] Karras T., Laine S., Aila T. A style-based generator architecture for generative adversarial networks.

[37] Wang S., Wang X., Hu Y. (2020). Diabetic retinopathy diagnosis using multichannel generative adversarial network with semisupervision. *IEEE Transactions on Automation Science and Engineering*.

[38] Yang T., Wu T., Li L., Zhu C. (2020). SUD-GAN: deep convolution generative adversarial network combined with short connection and dense block for retinal vessel segmentation. *Journal of Digital Imaging*.

[39] Sun K., Xiao B., Liu D., Wang J. Deep high-resolution representation learning for human pose estimation.

[40] Wang T. C., Liu M. Y., Zhu J. Y., Tao A., Kautz J., Catanzaro B. High-resolution image synthesis and semantic manipulation with conditional GANs.

[41] Hadsell R., Chopra S., LeCun Y. Dimensionality reduction by learning an invariant mapping.

[42] Ioffe S., Szegedy C. Batch normalization: accelerating deep network training by reducing internal covariate shift.

[43] Simonyan K., Zisserman A. (2014). Very deep convolutional networks for large-scale image recognition. https://arxiv.org/abs/1409.1556.

[44] Zhou Y., Wang B., Huang L., Cui S., Shao L. (2020). A benchmark for studying diabetic retinopathy: segmentation, grading, and transferability. *IEEE Transactions on Medical Imaging*.

[45] Porwal P., Pachade S., Kamble R. (2018). Indian diabetic retinopathy image dataset (IDRiD): a database for diabetic retinopathy screening research. *Data*.

[46] Staal J., Abramoff M. D., Niemeijer M., Viergever M. A., Van Ginneken B. (2004). Ridge-based vessel segmentation in color images of the retina. *IEEE Transactions on Medical Imaging*.

[47] Ronneberger O., Fischer P., Brox T. U-net: convolutional networks for biomedical image segmentation.

[48] Zhao H., Shi J., Qi X., Wang X., Jia J. Pyramid scene parsing network.

[49] Chen L.-C., Papandreou G., Schroff F., Adam H. (2017). Rethinking a trous convolution for semantic image segmentation. https://arxiv.org/abs/1706.05587.

[50] Heusel M., Ramsauer H., Unterthiner T., Nessler B., Hochreiter S. GANs trained by a two time-scale update rule converge to a local nash equilibrium.

[51] Szegedy C., Vanhoucke V., Ioffe S., Shlens J., Wojna Z. Rethinking the inception architecture for computer vision.

[52] Isola P., Zhu J. Y., Zhou T., Efros A. A. Image-to-Image translation with conditional adversarial networks.

[53] He K., Zhang X., Ren S., Sun J. Deep residual learning for image recognition.

[54] Lin Z., Guo R., Wang Y. A framework for identifying diabetic retinopathy based on anti-noise detection and attention-based fusion.

