# Supplementary material for: RF-GANs: A Method to Synthesize Retinal Fundus Images Based on Generative Adversarial Network
Source: Comput Intell Neurosci. 2021 Nov 10;2021:3812865. doi: 10.1155/2021/3812865 (PMC8598326; doi:10.1155/2021/3812865)
Supplement: Supplementary Materials — Quantitative Evaluation of RF-GAN1. [file 3812865.f1.docx]

# Supplementary Materials

## Quantitative Evaluation of RF-GAN1

### Segmentation of DR related lesions

To quantitatively evaluate the enhancement of RF-GAN1 for segmentation models to perform cross-domain DR-related lesion segmentation, IDRiD serves as source domain and FGADR as target domain. CycleGAN and RF-GAN1 are utilized to translate IDRiD respectively. Then the translated IDRiD acts as training set to train the HR-Net and the testing set of FGADR’s Seg-set acts as testing set to evaluate the segmentation performance of the model to assess the domain adaption performance of CycleGAN and RF-GAN1 respectively. The results are shown in Table 1.

Table 1: Performance evaluation of HR-Net trained by different datasets.

|  | Microaneurysms | | Hemorrhages | | Hard Exudates | | Soft Exudates | |
| --- | --- | --- | --- | --- | --- | --- | --- | --- |
|  | IOU | kappa | IOU | kappa | IOU | kappa | IOU | kappa |
| No | 0.5024 | 0.0139 | 0.5270 | 0.1256 | 0.4148 | 0.0272 | 0.5058 | 0.0308 |
| CycleGAN | 0.5025 | 0.0140 | 0.5118 | 0.0718 | 0.5195 | 0.1324 | 0.5209 | 0.0869 |
| RF-GAN1 | 0.5223 | 0.0912 | 0.6023 | 0.5212 | 0.5614 | 0.2386 | 0.5463 | 0.1623 |
| FGADR | 0.5832 | 0.1453 | 0.7042 | 0.5853 | 0.7122 | 0.6005 | 0.6211 | 0.3933 |

In Table 1, No denotes the HR-Net trained directly by IDRiD, and FGADR denotes the HR-Net trained by the training set of FGADR. CycleGAN and RF-GAN1 denote the HR-Net trained by IDRiD translated by CycleGAN and RF-GAN1 respectively. It can be seen from the Table 1: (1) The segmentation performance of HR-Net trained directly by IDRiD is poor, which implies substantial domain gap between different datasets. (2) The segmentation performance of HR-Net trained by IDRiD translated by CycleGAN dose not significantly improve compared to the HR-Net trained by the original IDRiD dataset. The segmentation performance of microaneurysms almost dose not improve, and the segmentation performance of hemorrhages decreases. The IOU and kappa for the segmentation of soft exudates, hard exudates increase by 0.0151 and 0.0561, 0.1047 and 0.1052 respectively. There is no much performance improvement in CycleGAN may be due to the fact that the generator is stuck into local minimum. (3) Compared with the HR-Net trained directly by IDRiD, the segmentation performance of the HR-Net trained by IDRiD translated by RF-GAN1 significantly improves. The IOU and kappa for the segmentation of microaneurysms, hemorrhages, hard exudates and soft exudates increase by 0.0199 and 0.0773, 0.0753 and 0.3956, 0.1466 and 0.2114, 0.0405 and 0.1315, respectively. The explanation is that the introduction of SiaNet and identity loss avoid RF-GAN1 falling into local minimum, so that RF-GAN1 can perform style transfer well while maintaining local details of the retinal fundus image. (4) Although the segmentation performance of HR-Net trained by IDRiD translated by RF-GAN1 improves, there is still a gap with HR-Net trained directly by FGADR. This may be due to two reasons. Firstly, there are so few images in IDRiD that HR-Net can’t get fully trained, resulting poor segmentation performance on the testing set of FGADR. Secondly, the imbalance between FGADR and IDRiD leads to the insignificant domain adaption performance of RF-GAN1. Therefore, in the process of translating images from segmentation datasets to EyePACS, we conduct clipping, flipping and rotation on segmentation datasets and undersampe EyePACS.

### Segmentation of vessels

To evaluate the performance of combining RF-GAN1 with HR-Net for cross-domain vessel segmentation on retinal fundus images, we translate the images from DRIVE to the domain of HRF using CycleGAN and RF-GAN1 respectively. Then we use original DRIVE dataset, DRIVE dataset translated by CycleGAN and RF-GAN1 to train HR-Net, the combination of TPCNN, DLBSVM and Firefly model [1]-[2] (We call TDF for simplicity here) and ASKFCM [3] Finally, we evaluate the vessel segmentation performance of models trained by different datasets on HRF [4]. The results are shown in Table 2.

Table 2: Performance comparisons of vessel segmentation with the competitive methods.

| Methods | Sensitivity | Specificity | Accuracy | IOU |
| --- | --- | --- | --- | --- |
| TDF | 0.7029 | 0.9913 | 0.9857 | 0.6815 |
| ASKFCM | 0.6524 | 0.9902 | 0.9842 | 0.6423 |
| HR-Net | 0.7322 | 0.9919 | 0.9864 | 0.7002 |
| TDF-r | 0.7381 | 0.9918 | 0.9864 | 0.6998 |
| ASKFCM-r | 0.6885 | 0.9908 | 0.9849 | 0.6610 |
| HR-Net-c | 0.7604 | 0.9924 | 0.9871 | 0.7104 |
| HR-Net-r | 0.7978 | 0.9930 | 0.9879 | 0.7305 |

XXX-r: segmentation models trained by DRIVE translated by RF-GAN1; XXX-c: segmentation models trained by DRIVE translated by CycleGAN

It can be seen from Table 2: (1) The vessel segmentation performance of models (TDF, ASKFCM and HR-Net) trained directly by DRIVE is not ideal, indicating great domain gap between DRIVE and HRF. (2) The sensitivity and IOU of HR-Net trained directly by DRIVE are higher than those of TDF and ASKFCM by 0.0293, 0.0187 and 0.0798, 0.0579 respectively, indicating that the cross-domain fundus vessel segmentation ability of HR-Net is stronger than that of TDF and ASKFCM. We speculate that this is due to two reasons. Firstly, HR-Net learns more domain-invariant features during training for vessel segmentation. Secondly, HR-Net connects high-to-low resolution subnetworks in parallel, so it can maintain high resolution of the extracted features of the retinal fundus images, extracting fine vessels at bifurcation point well. (3) The sensitivity and IOU of TDF, ASKFCM and HR-Net trained on the DRIVE dataset translated by RF-GAN1 increase by 0.0352, 0.0183, 0.0361, 0.0187 and 0.0656, 0.0303 when testing on HRF dataset. This proves that using RF-GAN1 can reduce the domain difference between source and target domains and improve the segmentation performance of fundus vessels. (4) The sensitivity, IOU, accuracy and specificity of HR-Net trained by the DRIVE dataset translated by RF-GAN are higher than those of HR-Net trained by the DRIVE dataset translated by CycleGAN, which shows that the domain adaption performance of RF-GAN1 is better than that of CycleGAN. (5) The combination of RF-GAN1 and HR-Net for cross-domain vessel segmentation achieves better performance than the competitive methods. Through the above experiments, we conclude that we are correct in choosing HR-Net, which is more capable of cross-domain segmentation, as the segmentation model and RF-GAN1, which is more capable of domain adaptation, as the style transfer model. Combining the two for cross-domain fundus vessel segmentation can achieve the desired performance.

1. Jebaseeli, T. Jemima, C. Anand Deva Durai, and J. Dinesh Peter, "Segmentation of retinal blood vessels from ophthalmologic diabetic retinopathy images," *Computers & Electrical Engineering*, vol. 73, pp. 245-258, 2019.
2. Jebaseeli, T. Jemima, C. Anand Deva Durai, and J. Dinesh Peter, "Retinal blood vessel segmentation from diabetic retinopathy images using tandem PCNN model and deep learning based SVM," *Optik*, vol. 199, pp. 163328, 2019.
3. Jebaseeli, T. Jemima, C. Anand Deva Durai, and J. Dinesh Peter, "IOT based sustainable diabetic retinopathy diagnosis system," *Sustainable Computing: Informatics and Systems*, vol. 28, pp. 100272, 2020.
4. Budai, Attila, et al, "Robust vessel segmentation in fundus images," *International journal of biomedical imaging*, vol. 2013, 2013.
